# Changes in actual arm-hand use in stroke patients during and after clinical rehabilitation involving a well-defined arm-hand rehabilitation program: A prospective cohort study

**DOI:** 10.1371/journal.pone.0214651

**Published:** 2019-04-01

**Authors:** Johan Anton Franck, Rob Johannes Elise Marie Smeets, Henk Alexander Maria Seelen

**Affiliations:** 1 Adelante Rehabilitation Centre, dept. of Brain Injury Rehabilitation, Hoensbroek, the Netherlands; 2 Adelante Centre of Expertise in Rehabilitation and Audiology, Hoensbroek, the Netherlands; 3 Maastricht University, Research School CAPHRI, dept. of Rehabilitation Medicine, Maastricht, the Netherlands; Vanderbilt University, UNITED STATES

## Abstract

**Introduction:**

Improvement of arm-hand function and arm-hand skill performance in stroke patients is reported by many authors. However, therapy content often is poorly described, data on actual arm-hand use are scarce, and, as follow-up time often is very short, little information on patients’ mid- and long-term progression is available. Also, outcome data mainly stem from either a general patient group, unstratified for the severity of arm-hand impairment, or a very specific patient group.

**Objectives:**

To investigate to what extent the rate of improvement or deterioration of actual arm-hand use differs between stroke patients with either a severely, moderately or mildly affected arm-hand, during and after rehabilitation involving a well-defined rehabilitation program.

**Methods:**

Design: single–armed prospective cohort study. Outcome measure: affected arm-hand use during daily tasks (accelerometry), expressed as ‘Intensity-of arm-hand-use’ and ‘Duration-of-arm-hand-use’ during waking hours. Measurement dates: at admission, clinical discharge and 3, 6, 9, and 12 months post-discharge. Statistics: Two-way repeated measures ANOVAs.

**Results:**

Seventy-six patients (63 males); mean age: 57.6 years (sd:10.6); post-stroke time: 29.8 days (sd:20.1) participated. Between baseline and 1-year follow-up, Intensity-of-arm-hand-use on the affected side increased by 51%, 114% and 14% (p < .000) in the mildly, moderately and severely affected patients, respectively. Similarly, Duration-of-arm-hand-use increased by 26%, 220% and 161% (p < .000). Regarding bimanual arm-hand use: Intensity-of-arm-hand-use increased by 44%, 74% and 30% (p < .000), whereas Duration-of-arm-hand-use increased by 10%, 22% and 16% (p < .000).

**Conclusion:**

Stroke survivors with a severely, moderately or mildly affected arm-hand showed different, though (clinically) important, improvements in actual arm-hand use during the rehabilitation phase. Intensity-of-arm-hand-use and Duration-of-arm-hand-use significantly improved in both unimanual and bimanual tasks/skills. These improvements were maintained until at least 1 year post-discharge.

## Introduction

After stroke, the majority of stroke survivors experiences significant arm-hand impairments [1, 2] and a decreased use of the paretic arm and hand in daily life [3]. The actual use of the affected hand in daily life performance depends on the severity of the arm-hand impairment [4–6] and is associated with perceived limitations in participation [7, 8]. Severity of arm-hand impairment is also associated with a decrease of health-related quality of life [9], restricted social participation [10], and subjective well-being [11, 12].

Numerous interventions and arm-hand rehabilitation programs have been developed in order to resolve arm-hand impairments in stroke patients [6, 13]. In the Netherlands, a number of stroke units in rehabilitation centres implemented a well-described ‘therapy-as-usual’ arm-hand rehabilitation program, called CARAS (acronym for: Concise Arm and hand Rehabilitation Approach in Stroke)[14], serving a broad spectrum of stroke patients across the full stroke severity range of arm-hand impairments. The arm-hand rehabilitation program has been developed to guide clinicians in systematically designing arm-hand rehabilitation, tailored towards the individual patient’s characteristics while keeping control over the overall heterogeneity of this population typically seen in stroke rehabilitation centres. A vast majority of stroke patients who participated in CARAS improved on arm-hand function (AHF), on arm-hand skilled performance (AHSP) capacity and on (self-) perceived performance, both during and after clinical rehabilitation [15]. The term ‘arm-hand function’ (AHF) refers to the International Classification of Functioning (ICF) [16] ‘*body function and structures level’*. The term ‘arm-hand skilled performance’ (AHSP) refers to the ICF ‘*activity level’*, covering capacity as well as both perceived performance and actual arm-hand use [17].

Improved AHF and/or AHSP capacity do not automatically lead to an increase in actual arm-hand use and do not guarantee an increase of performing functional activities in daily life [18–20]. Improvements at function level, i.e. regaining selectivity, (grip) strength and/or grip performance, do *not* automatically lead to improvements experienced in real life task performance of persons in the post-stroke phase who live at home [18, 21]. Next to outcome measures regarding AHF, AHSP capacity and (self-) perceived AHSP, which are typically measured in controlled conditions, objective assessment of functional activity and actual arm-hand use outside the testing situation is warranted [22, 23].

Accelerometry can be used to reliably and objectively assess actual arm-hand use during daily task performance [24–32]and has been used in several studies to detect arm-hand movements and evaluate arm-hand use in the post-stroke phase [20, 33–35]. Previous studies have demonstrated that, in stroke patients, movement counts, as measured with accelerometers, are associated with the use of the affected arm-hand (Motor Activity Log score) [36, 37] and, at function level, with the Fugl-Meyer Assessment [38]. Next to quantifying paretic arm-hand use, accelerometers have also been used to provide feedback to further enhance the use of the affected hand in home-based situations [39]. Most studies consist of relatively small [27, 30, 40–44] and highly selected study populations [45] with short time intervals between baseline and follow-up measurements. As to our knowledge, only a few studies monitored arm-hand use in stroke patients for a longer period, i.e. between time of discharge to a home situation or till 6 to 12 months after stroke [19, 44, 46]. However, they used a relatively small study sample and their intervention aimed at arm-hand rehabilitation was undefined. Both studies of Connell et al. and Uswatte et al. describe a well-defined arm hand intervention where accelerometry data were used as an outcome measure [27, 47]. However, the study population described by Connell et al. consisted of a relative small and a relative mildly impaired group of chronic stroke survivors. The study population described by Uswatte et al. consisted of a large group of sub-acute stroke patients within strict inclusion criteria ranges [37], who, due to significant spontaneous neurologic recovery within this sub-acute phase, had a mildly impaired arm and hand [48, 49]. This means that the group lacked persons with a moderately to severely affected arm-hand, who are commonly treated in the daily rehabilitation setting.

The course of AHF and AHSP of a broad range of sub-acute stroke patients during and after rehabilitation involving a well-defined arm-hand rehabilitation program (i.e. CARAS) [14] has been reported by Franck et al. [15]. The present paper provides data concerning actual arm-hand use in the same study population, and focuses on two objectives. The first aim is to investigate changes in actual arm-hand use across time, i.e. during and after clinical rehabilitation, within a stroke patient group typically seen in daily medical rehabilitation practice, i.e. covering a broad spectrum of arm-hand problem severity levels, who followed a well-described arm-hand treatment regime. The second aim is to investigate to what extent improvement (or deterioration) regarding the use of the affected arm-hand in daily life situations differs between patient categories, i.e. patients with either a severely, moderately or mildly impaired arm-hand, during and after their rehabilitation, involving a well-defined arm-hand rehabilitation program.

## Methods

### Design

This study is a single-armed prospective cohort study conducted between February 2011 and May 2015. Stroke patients who experienced AHF impairments were assessed during and up till 12 months after their protocolled rehabilitation treatment. This study meets the principles as stated in the Declaration of Helsinki and was approved by the Medical Ethics Committee of Maastricht University Medical Centre in the Netherlands (dossier number NL35681.068.11).

Written informed consent was obtained from all participants prior to the start of their participation in this study.

### Population

The study population consisted of a broad range of sub-acute stroke patients admitted to the inpatient stroke ward of Adelante Rehabilitation Centre in Hoensbroek, the Netherlands. Inclusion criteria were kept to a minimum, i.e.: age ≥18 years; clinically diagnosed with central paresis of the arm/hand at entry in the study; ability to control sitting posture; a fair cognitive level, i.e. being able to understand the questionnaires and measurement instructions. Exclusion criteria were: additional complaints that may interfere with the execution of the measurements; no informed consent.

### Procedures

#### Therapy-as-usual

After having been admitted to the Concise Arm and hand Rehabilitation Approach in Stroke (CARAS) [14], all participants were coached in arm-hand training, how to cope with their affected hand and to (re-)explore how to use their arm-hand in daily life activities or, in case dexterity was lacking completely, how to learn to keep and maintain the affected arm-hand in an optimal condition. CARAS targets a broad spectrum of arm-hand impairments typically seen in a heterogeneous stroke rehabilitation population. This approach consists of a well-described program offering stepwise, transparent and comprehensible procedures, tailored to specific needs of the individual patient.

Based on the severity of arm–hand impairment, for which the Utrechtse Arm-hand Test (UAT) was used [50], patients were stratified into three subgroups, i.e. subgroup 1 (severely affected arm-hand (UAT score 0–1)), subgroup 2 (moderately affected arm-hand (UAT score 2–3)), and subgroup 3 (mildly affected arm-hand (UAT score 4–7)). All patients followed one of three training programs within CARAS. Subgroup 1 followed program 1, titled ‘*taking care and prevention’*. These patients are unable to use their affected arm and hand for skill performance in daily life situations (non-functional arm-hand). Program 1 contains different topics aimed at getting and keeping the affected shoulder and arm-hand in an optimal condition and learning strategies on what to do when discomfort arises. Patients in subgroup 2 (UAT score 2–3) were admitted to program 2, whereas patients in subgroup 3 (UAT score 4–7) followed program 3. Both program 2 and 3 incorporate (*high) intensive*, *task-oriented training components* aimed at optimal integration of the affected arm and hand in daily occupations. Patients in subgroup 2 work on passive and active stabilisation tasks in order to become able to use their affected arm and hand for, e.g., holding vegetables on a table while cutting them with a knife held in the non-affected hand. Patients from subgroup 3 relearn their abilities to use their affected arm and hand instantaneously in daily situations in which more complex (bi-)manual activities are necessary. Patients in program 1 spend 4.5 hours per week on training during a period of six weeks. Patients in program 2 spend six hours of training per week during 12 weeks and patients in program 3 spend six hours of training during each week for 6 weeks. In this study CARAS is considered as ‘therapy-as-usual’ i.e. the standard rehabilitation approach for patients who cope with arm-hand deficits due to stroke occurrence. A more detailed description of the therapy content and the basic assumptions of CARAS have been presented elsewhere by Franck and co-workers [14].

After baseline assessment, patients enrolled in one of the three programs and started training for six consecutive weeks. After six weeks the patient left the program and entered the second assessment. Progress made, was expressed in terms of functional goals reached, based on capacity and performance levels exceeding certain minimal clinically important thresholds, as captured by the outcome measures at function level and activity level. Depending on these results, it was possible for the patient to choose for a second (and final) six weeks period of training, which was then also evaluated [14].

#### Outcome measures

At the start of the study the following demographic data and characteristics of participants were recorded: Gender, age, time post-stroke, stroke localisation, affected side, hand dominance, and status of the affected arm and hand expressed in terms of dexterity (severely, moderately or mildly affected), based on the UAT score [50].

Every two weeks a short question was posed in order to establish the occurrence of any major event that may have prevented the participant from using one or both arms or hands. The questions posed, was: “Has there been any major problem during the last two weeks preventing you from using one or both hands? (yes/no). If so, please indicate (from a short list) which problem(s).”This information is important as it may be used in explaining changes in data trends due to non-therapy-related events (e.g. sudden reduction in arm-hand use because the patient became ill and was confined to bed).

At each measurement time point the amount of arm-hand use (on both the affected and non-affected side) was monitored for 3 consecutive days, using watch-like accelerometers (Actiwatches (Actiwatch AW7, CamNtech, UK)). Accelerations were recorded and converted into digital signals with a sample frequency of 32 Hz and a quantization range between -128 and +128. Accelerations between -5g and +5g can be recorded by the system. The threshold of movement detection (= system sensitivity) is 0.05g. [36]. Per measurement session, two Actiwatches had to be worn, one on each wrist. Both Actiwatches were held in place using a nylon strap on the wrist, i.e. at the distal part of the radius, with the device facing the dorsal side of the forearm.

#### Measurement dates

**Fig 1:**
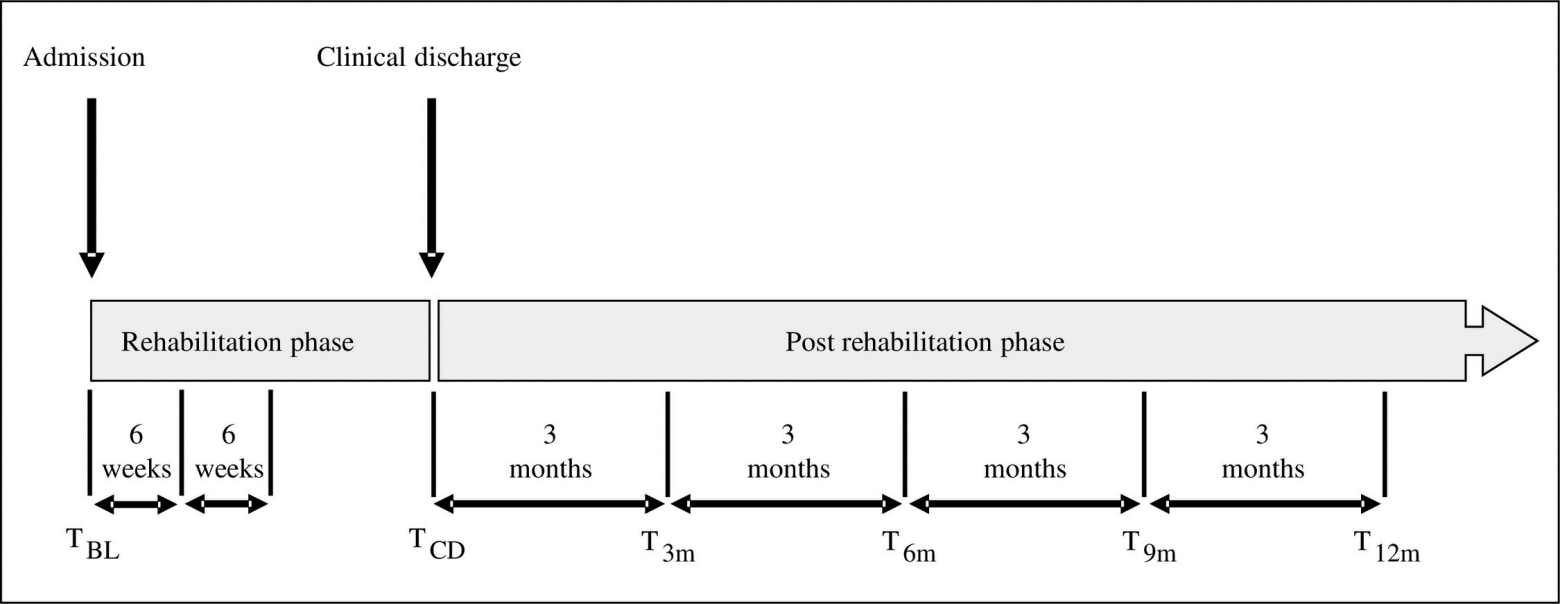
Overview of measurement timing. T = time; BL = baseline; CD = clinical discharge; M = month.

This study included six measurements. After admission to the rehabilitation centre and prior to the start in CARAS a baseline measurement (T_BL_) was performed, followed by a measurement at clinical discharge (T_CD_). After discharge, measurements were performed at four additional points in time, interspaced by three months: T_3m_ (= T_CD_ + 3 months) through T_12m_ (= T_CD_ + 12 months). Per measurement session, the participants wore both Actiwatches for a period of three consecutive days in which the amount of arm-hand use was recorded continuously. Fig 1 presents an overview of the measurement dates.

#### Signal processing

Accelerations caused by arm-hand movement were recorded as analogue signals which were subsequently converted into a digital signal. The highest amplitude per 1-second period, representing peak intensity, was registered as a “count”. Every two seconds the two peak intensity values identified, were summed into a single number. This 2-second period was called an epoch [32, 51]. In this study the duration of the epoch was set per 2 seconds. This number then became a single data point in the final outcome signal or ‘count’ time series [36].

**Fig 2:**
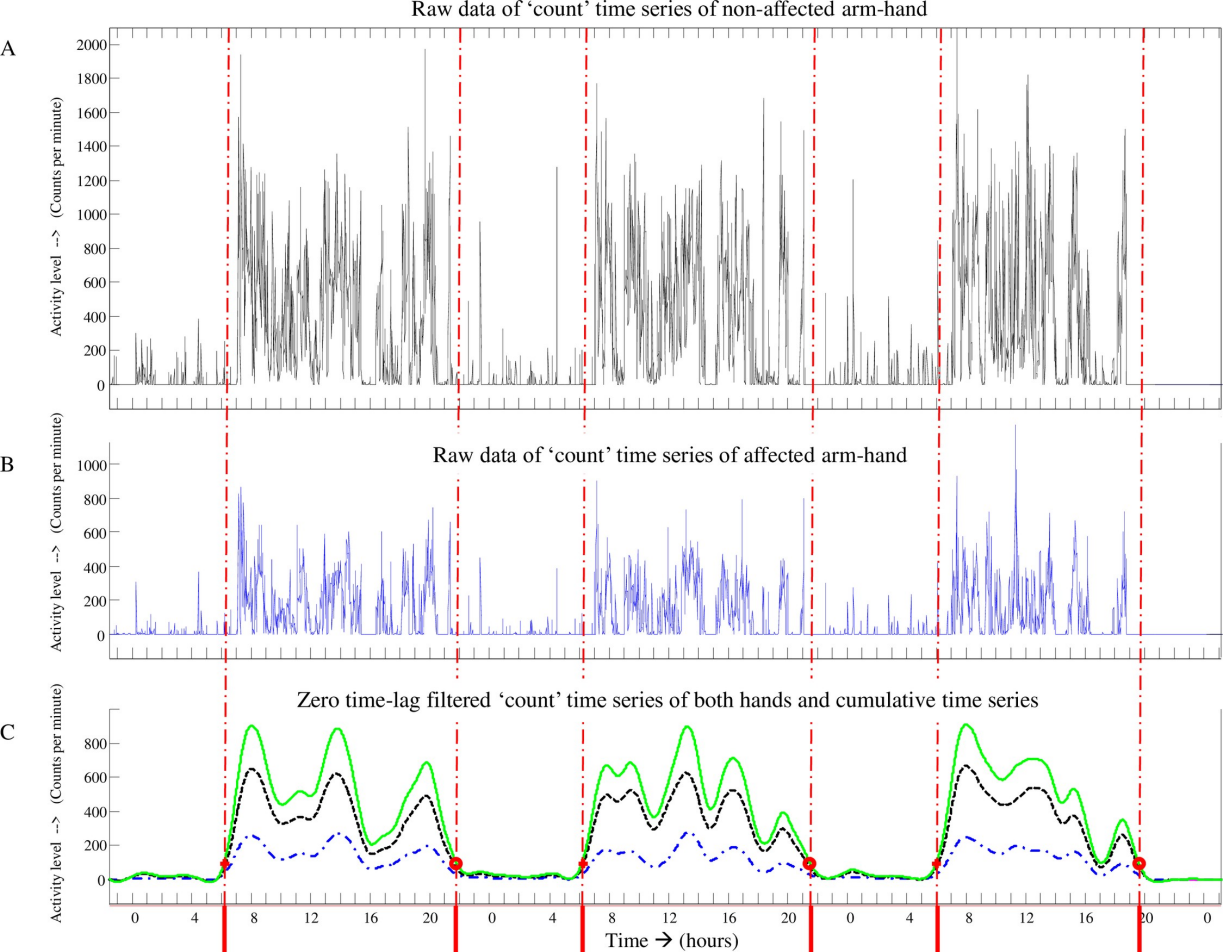
Accelerometer data ‘count’ time series of three consecutive days. A = ‘count’ time series from the non-affected arm; B = count’ time series from the affected arm; Dotted time series in Fig 2C = zero time-lag filtered signal from non-affected arm; Dashed-dotted time series in Fig 2C = zero time-lag filtered signal from affected arm; Solid time series in Fig 2C = cumulative signal representing both filtered signals; vertical dashed-dotted lines = start and end of the so-called ‘uptime’ per day.

Fig 2 provides an example of a ‘count’ time series of three consecutive days of accelerometry data from a participant suffering from a paresis of the arm/hand.

Fig 2: Fig 2A represents data from the non-affected arm-hand, whereas Fig 2B represents data from the affected arm-hand. Fig 2C represents the zero time-lag low pass filtered signal of the non-affected arm-hand (dotted time series), the affected arm-hand (dashed-dotted time series) and the cumulative signal of the data of both hands (solid time series).

First, both ‘count’ time series, from the non-affected and the affected arm (‘A’ and ‘B’ respectively in Fig 2), were filtered using a zero time-lag second-order Butterworth filter (cut-off frequency: 0.0025 Hz) (dotted time series and dashed-dotted time series in Fig 2C) and subsequently cumulated into one signal (solid line in Fig 2C). From the latter signal, representing the arm-hand activity of both sides, the start and end of the so-called ‘*uptime*’ per day was identified (delimited by the vertical dashed-dotted lines on the time axis in Fig 2), using a predefined threshold. In order to avoid small resting periods during the uptime, in which neither arm-hand was used, to be falsely detected as ‘night time’, night time was a-priori defined as being longer than four consecutive hours. Minimal uptime length was defined as, at least, 10 consecutive hours per day. Night time data were discarded.

Next, for each uptime, the average sum of ‘counts’ per minute was calculated for each of the two devices, i.e. for both arm-hands separately. This average sum of ‘counts’ per minute represents *‘Intensity-of-arm-hand-use’* of each arm-hand during the uptime period per minute [36] [51]. ‘*Duration-of-arm-hand-use’* was calculated as the total amount of time (in seconds) in which there was activity of one or both hands exceeding a minimal threshold of signal intensity during the uptime.

Subsequently, more specific information on Intensity-of-arm-hand-use and on Duration-of-arm-hand-use of both the affected and non-affected arm-hand was extracted from the ‘count’ time series data collected during uptime. First, the time (during uptime) in which the affected arm-hand was used unimanually, the time in which the non-affected arm-hand was used unimanually, the time both hands were used and the time none of the hands were used, was calculated, based on signal intensity crossing a predefined threshold. Next, for each of the aforementioned times the Intensity-of-arm-hand-use was calculated.

Finally, in order to compare the Intensity-of-arm-hand-use of the affected arm-hand to that of the non-affected arm-hand, the ratio between the sum of counts of the affected arm (numerator) and the unaffected arm (denominator) was calculated. To avoid underestimation of the non-impaired arm-hand counts (denominator), compared to the impaired arm-hand (numerator), this ratio was log-transformed before (sub-)group averages were calculated [36]. Similarly, this procedure was done for the Duration-of-arm-hand-use data.

All accelerometry data were analyzed using MATLAB software version R2016a (The MathWorks Inc, Natick, MA, USA) and Microsoft Excel software version 2010 (Microsoft Corporation, Redmond, Washington, USA).

#### Handling of missing values

In order to handle missing data we used the following four decision rules, formulated prior to the start of the study;

1. When the baseline value was missing, this value was estimated using the mean baseline value of the subgroup the patient was allocated to.
2. When the T_12m_ value was missing, the ‘last observation carried forward’ procedure was used [52].
3. When 1 or 2 (temporally adjacent) value(s), not being the baseline value or the T_12m_ value, were missing, these missing value(s) were estimated by linear interpolation using the two valid adjacent values in the time series.
4. In case of 3 or more missing values, the whole case was discarded.

#### Statistical analyses

The statistical analysis was performed using the intention-to-treat approach, following up all participants as originally allocated to a particular subgroup (1, 2 or 3) of CARAS.

Statistical analyses included two-way repeated measures Analysis of Variance (ANOVA) (within-subject factor: *Time*, between-subject factor: *AHF status*). To test for normal distribution, the Shapiro-Wilk test was used [53]. Subsequently, multiple comparison was done using a Bonferroni approach to control for spurious false positive findings, involving two combinations of data sets, i.e. T_BL_ vs. T_CD_ and T_CD_ vs. T_12m_, representing the clinical rehabilitation phase, and the post-rehabilitation phase, respectively. Alpha was set at 0.05. Data were analyzed using SPSS software (version 24.0) (IBM Inc. Armonk, NY).

In the results section below, accelerometry data with respect to both Intensity-of-arm-hand-use and Duration-of arm-hand-use are reported in the following order: First, results regarding the affected arm-hand are presented. Second, the results of the use of the affected arm-hand during unimanual activities, are presented. Third, results regarding the use of both hands, while performing a task bimanually, are presented. And four, data regarding the ratio of Intensity-of-arm-hand-use of the affected hand and for both hands are presented.

## Results

### Patient characteristics and error analysis

A total of 89 patients entered the study. As a result of the application of the four previously stated decision rules, the data of 13 participants were discarded (rule number 4), leading to a remaining group of 76 participants used in the statistical analysis. For 16 (out of 380) measurement points, data were interpolated in accordance with rules 2 and 3.

Patient characteristics are presented in Table 1.

**Table 1:** Overview of patient characteristics at baseline.

Characteristics	Whole group	Subgroups		
		Gr1	Gr2	Gr3
Total number: (n)	89	28	28	33
Age: (years) (mean +/-sd)	57.6 +/-10.6	56.2 +/-11.0	57.9 +/- 12.5	58.5 +/- 8.5
Gender: Male (n (%)) Female (n (%))	63 (70.8%) 26 (29.2%)	15 (53.6%) 13 (46.4%)	24 (85.7%) 4 (14.3%)	24 (72.7%) 9 (27.3%)
Lesion side: Left (n (%)) Right (n (%))	53 (59.6%) 36 (40.4%)	13 (46.4%) 15 (53.6%)	18 (64.3%) 10 (35.7%)	22 (66.7%) 11 (33.3%)
Stroke Type: Haemorrhagic (n (%)) Ischemic (n (%))	17 (19.1%) 72 (80.9%)	5 (17.9%) 23 (82.1%)	5 (17.9%) 23 (82.1%)	7 (21.2%) 26 (78.8%)
Time post stroke: (days) (mean +/-sd)	29.8 +/-20.1	40 +/-27.5	27 +/-14.5	23.4 +/-12.6
Affected hand: Dominant (n(%)) Non-dominant (n (%))	50 (56.2%) 39 (43.8%)	11 (39.3%) 17 (60.7%)	17 (60.7%) 11 (39.3%)	22 (66.6%) 11 (33.3%)
Lesion site (n)	Basal ganglia (7), Brainstem (2), Capsula interna (1), Cerebellum (2), Frontal area (2), Frontoparietal area (1), Frontotemporal area (2), Parietal area (1), Parietotemporal area (1), Posterior area (1), Temporal area (1), Temporal area & thalamus (1), Thalamus (4), Pontine (1), Hemispheric (not specified) (54), Lacunar (5), Medulla oblongata & cerebellum (1), Nucleus caudatus (2).			

#### Intensity-of-arm-hand-use on the affected side during (overall) uptime

**Fig 3:**
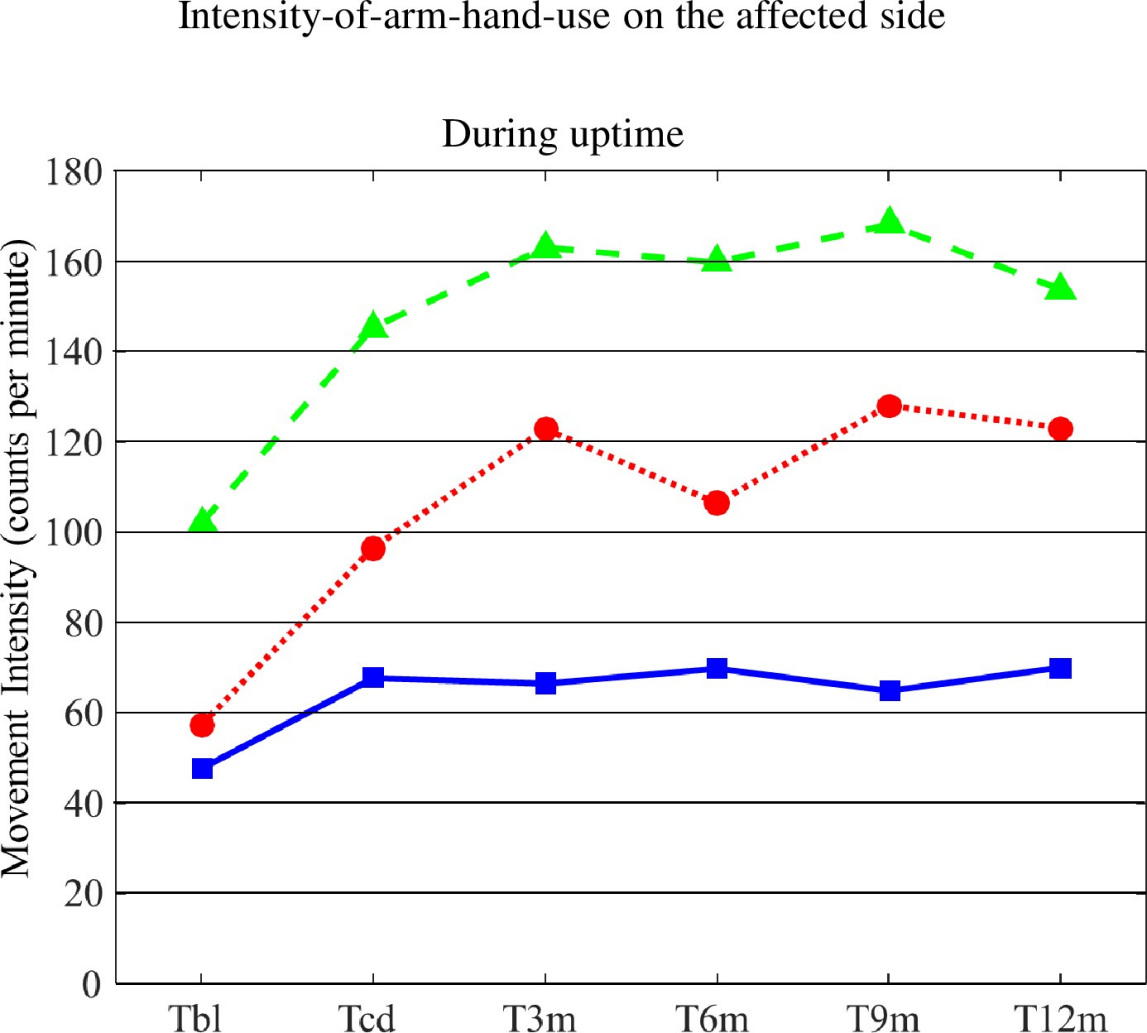
Mean values for Intensity-of-arm-hand-use during uptime for subgroups 1, 2 and 3. T = time; bl = baseline; cd = clinical discharge; m = month; Solid line = subgroup 1; Dotted line = subgroup 2; Dashed line = subgroup 3.

Mean values for Intensity-of-arm-hand-use during (overall) uptime for subgroups 1, 2 and 3 are displayed in Fig 3. The corresponding statistics are presented in Table 2.

**Table 2:** Statistics regarding Intensity-of-arm-hand-use.

				Overall uptime		Unimanual		Bimanual		
	Factor	Group	DF	F-value	p-value	F-value	p-value	F-value		p-value
**T**_**BL**_ **through T**_**12m**_										
Main effects	Time		5	13.474	0.000	6.707	0.000	23.555		0.000
	AHF status		2	13.906	0.000	4.180	0.019	13.717		0.000
Interaction	Time x AHF status		10	2.852	0.002	1.338	0.209	2.530		0.006
Post-hoc MC	AHF status	Gr1-Gr2			0.084		0.972			0.036
		Gr1-Gr3			0.000		0.017			0.000
		Gr2-Gr3			0.035		0.350			0.091
**T**_**BL**_ **versus T**_**CD**_										
Main effects	Time		1	28.271	0.000	13.033	0.001	30.727		0.000
	AHF status		2	14.852	0.000	3.498	0.036	12.860		0.000
Interaction	Time x AHF status		2	1.341	0.268	0.956	0.390	1.429		0.246
Post-hoc MC	AHF status	Gr1-Gr2			0.522		1.000			0.646
		Gr1-Gr3			0.000		0.045			0.000
		Gr2-Gr3			0.002		0.230			0.005
**T**_**CD**_ **versus T**_**12m**_										
Main effects	Time		1	6.396	0.014	0 .689	0.409	15.593		0.000
	AHF status		2	12.701	0.000	4.458	0.015	11.706		0.000
Interaction	Time x AHF status		2	1.934	0.152	0.385	0.682	2.055		0.135
Post-hoc MC	AHF status	Gr1-Gr2			0.075		0.359			0.065
		Gr1-Gr3			0.000		0.012			0.000
		Gr2-Gr3			0.069		0.763			0.124

MC = Multiple comparison; GR = subgroup: AHF = Arm-Hand Function; DF = degrees of freedom

From T_bl_ across to T_12m_ main significant effects for ‘Time’, ‘AHF status’ and ‘Time’ x ‘AHF status’ were found. Furthermore, whereas, on average, all 3 subgroups showed improvement between baseline and clinical discharge regarding Intensity-of-arm-hand-use on the affected side during uptime, participants from subgroup 2 seem to even further improve between discharge (T_CD_) and 12 months after clinical discharge (T_12m_). Participants admitted to subgroup 3 improved the most between baseline (T_BL)_ and clinical discharge (T_CD_).

#### Intensity-of-arm-hand-use on the affected side during unimanual activities

**Fig 4:**
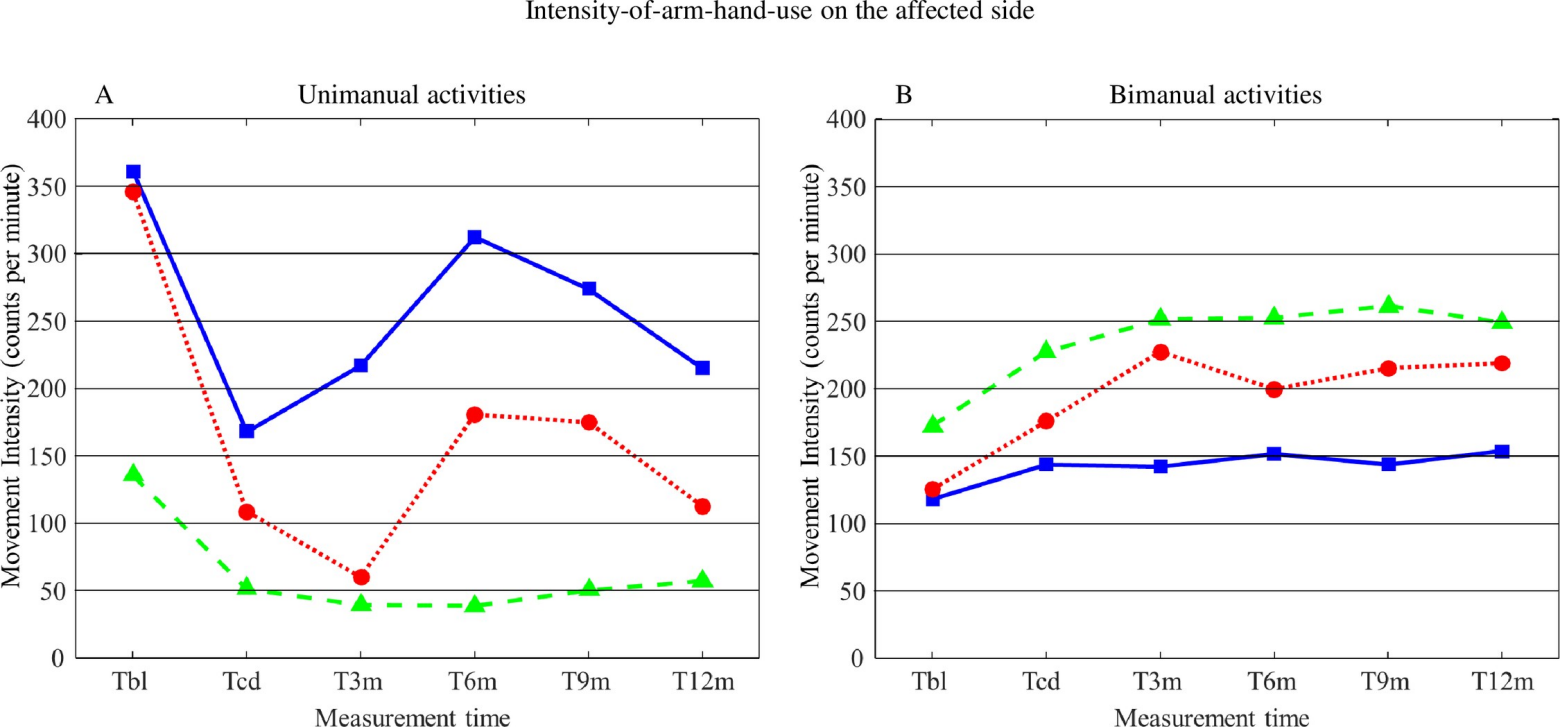
**A: Mean values for Intensity-of-arm-hand-use on the affected side during unimanual activities for subgroups 1, 2 and 3. B: Mean values for Intensity-of-arm-hand-use on the affected side during bimanual activities for subgroups 1, 2 and 3.** T = time; bl = baseline; cd = clinical discharge; m = month. Solid line = subgroup 1; Dotted line = subgroup 2; Dashed line = subgroup 3.

Mean values for Intensity-of-arm-hand-use on the affected side during unimanual activities for subgroups 1, 2 and 3 are displayed in Fig 4A. The corresponding statistics are presented in Table 2.

The main findings were that, on average, the Intensity-of-arm-hand-use on the affected side during unimanual activities decreased in subgroup 1 and 2, and, although not statistically significant, to a lesser extent, in subgroup 3, between T_BL_ and T_CD_. Regarding results from T_CD_ and T_12m_, no further change in Intensity-of-arm-hand-use on the affected side during unimanual activities was observed.

#### Intensity-of-arm-hand-use on the affected side during bimanual activities

Mean values for Intensity-of-arm-hand-use on the affected side during bimanual activities for subgroups 1, 2 and 3 are displayed in Fig 4B. The corresponding statistics are presented in Table 2.

During bimanual task performance Intensity-of-arm-hand-use on the affected side improved in all three subgroups across time, i.e. between T_BL_ and T_12m_. However, in subgroup 2 improvement between T_BL_ and T_12m_ was relatively larger compared to subgroup 1 and, though to a lesser extent, to subgroup 3. At baseline, participants from subgroup 2 used their affected arm-hand approximately equally to participants from subgroup 1 during bimanual activities.

#### Duration-of-arm-hand-use on the affected side during unimanual activities

**Fig 5:**
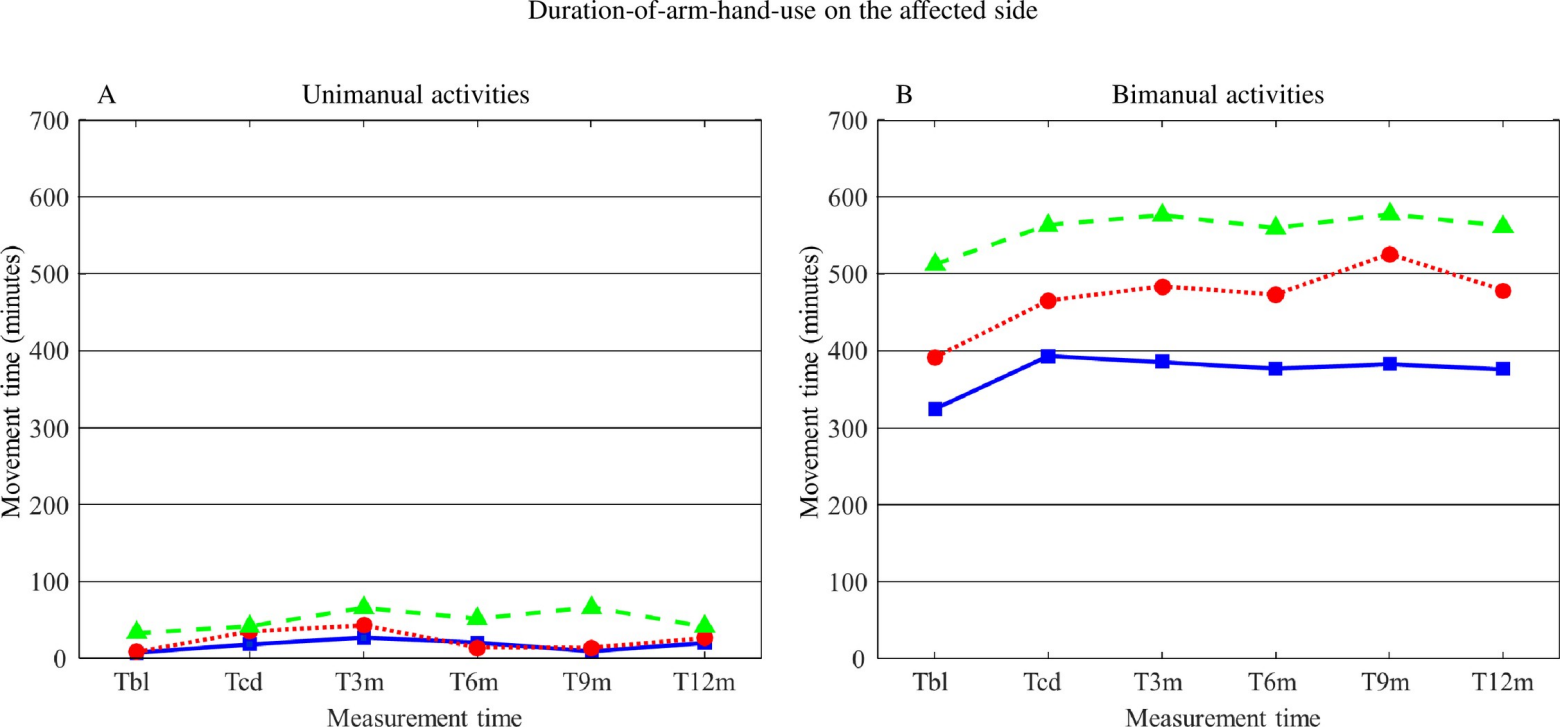
**A: Mean values for Duration-of-arm-hand-use on the affected side during unimanual activities for subgroups 1, 2 and 3. B: Mean values for Duration-of-arm-hand-use on the affected side during bimanual activities for subgroups 1, 2 and 3.** T = time; bl = baseline; cd = clinical discharge; m = month. Solid line = subgroup 1; Dotted line = subgroup 2; Dashed line = subgroup 3.

Mean values for Duration-of-arm-hand-use on the affected side during unimanual activities for subgroups 1, 2 and 3 are displayed in Fig 5A. The corresponding statistics are presented in Table 3.

**Table 3:** Statistics regarding Duration-of-arm-hand-use.

				Unimanual		Bimanual		
	Factor	Group	DF	F-value	p-value	F-value	p-value	
**T**_**BL**_ **through T**_**12m**_								
Main effects	Time		5	4.438	0.001	7.221	0.000	
	AHF status		2	12.510	0.000	14.258	0.000	
Interaction	Time x AHF status		10	1.545	0.122	0.539	0.862	
Post-hoc MC	AHF status	Gr1-Gr2			1.000		0.046	
		Gr1-Gr3			0.000		0.000	
		Gr2-Gr3			0.003		0.056	
**T**_**BL**_ **versus T**_**CD**_								
Main effects	Time		1	9.894	0.002	11.952	0.001	
	AHF status		2	6.224	0.003	13.238	0.000	
Interaction	Time x AHF status		2	1.220	0.301	0.152	0.859	
Post-hoc MC	AHF status	Gr1-Gr2			0.841		0.841	
		Gr1-Gr3			0.003		0.000	
		Gr2-Gr3			0.139		0.251	
**T**_**CD**_ **versus T**_**12m**_								
Main effects	Time		1	0.231	0.632	0 .015	0.903	
	AHF status		2	3.726	0.029	11.294	0.000	
Interaction	Time x AHF status		2	0.359	0.699	0.383	0.683	
Post-hoc MC	AHF status	Gr1-Gr2			0.640		0.124	
		Gr1-Gr3			0.024		0.000	
		Gr2-Gr3			0.654		0.078	

MC = Multiple comparison; GR = subgroup: AHF = Arm-Hand Function; DF = degrees of freedom

Although between T_bl_ and T_CD,_ and also from T_bl_ across to T_12m_ main significant effects for ‘Time’ and ‘AHF status’ were found regarding Duration-of-arm-hand-use on the affected side during unimanual activities, differences were rather small. Most distinct differences between subgroups at all points in time were found between subgroup 1 and 3.

#### Duration-of-arm-hand-use on the affected side during bimanual activities

Mean values for Duration-of-arm-hand-use on the affected side during bimanual activities for subgroups 1, 2 and 3 are also displayed in Fig 5B. The corresponding statistics are presented in Table 3.

Significant main effects for ‘Time’ and for ‘AHF status’ regarding Duration-of-arm-hand-use on the affected side during bimanual activities were found for the phase between T_BL_ and T_CD_ as well as for the phase between T_BL_ and T_12m_. Subgroup differences were most prominent between subgroup 1 and 3.

#### Mean ratios regarding Intensity of arm-hand use between the affected and the non-affected arm-hand during uptime

**Fig 6:**
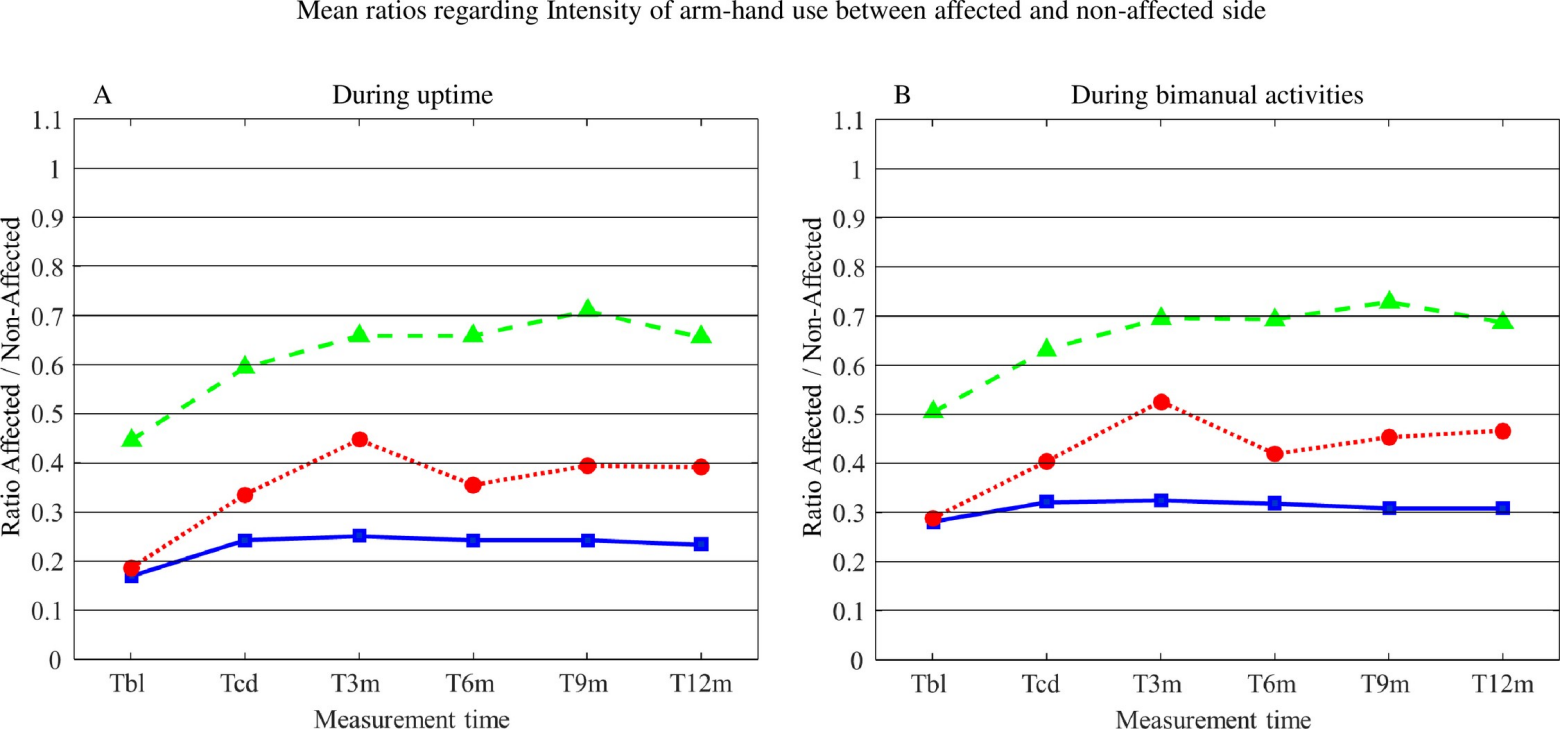
**A: Mean ratios regarding Intensity of arm-hand use between the affected and the non-affected arm-hand during uptime for subgroups 1, 2 and 3. B: Mean ratios regarding Intensity of arm-hand use between the affected and the non-affected arm-hand during bimanual activities for subgroups 1, 2 and 3.** T = time; bl = baseline; cd = clinical discharge; m = month; A = affected side; NA = non-affected side. Solid line = subgroup 1; Dotted line = subgroup 2; Dashed line = subgroup 3.

Mean ratios regarding Intensity of arm-hand use between the affected and the non-affected arm-hand during uptime for subgroups 1, 2 and 3 are displayed in Fig 6A. The corresponding statistics are presented in Table 4.

**Table 4:** Statistics regarding Intensity-of-arm-hand-use ratios between affected and non-affected arm-hand.

					During uptime			During bimanual use	
		Factor	Group	DF	F-value	p-value		F-value	p-value
**T**_**BL**_ **through T**_**12m**_									
Main effects	Time			5	24.729	0.000		7.221	0.000
	AHF status			2	39.088	0.000		14.258	0.000
Interaction	Time x AHF status			10	1.796	0.060		0.539	0.862
Post-hoc MC	AHF status		Gr1-Gr2			0.008			0.046
			Gr1-Gr3			0.000			0.000
			Gr2-Gr3			0.000			0.056
**T**_**BL**_ **versus T**_**CD**_									
Main effects	Time			1	38.731	0.000		11.952	0.001
	AHF status			2	34.127	0.000		13.238	0.000
Interaction	Time x AHF status			2	1.801	0.172		0.152	0.859
Post-hoc MC	AHF status		Gr1-Gr2			0.375			0.841
			Gr1-Gr3			0.000			0.000
			Gr2-Gr3			0.000			0.251
**T**_**CD**_ **versus T**_**12m**_									
Main effects	Time			1	1.045	0.196		0 .015	0.903
	AHF status			2	11.294	0.000		11.294	0.000
Interaction	Time x AHF status			2	1.045	0.357		0.383	0.683
Post-hoc MC	AHF status		Gr1-Gr2			0.007			0.124
			Gr1-Gr3			0.000			0.000
			Gr2-Gr3			0.000			0.078

MC = Multiple comparison; GR = subgroup: AHF = Arm-Hand Function; DF = degrees of freedom

During uptime the ratio of Intensity-of-arm-hand-use between the affected and non-affected side increased in all subgroups across the clinical and post-clinical period, with major gains observed between T_BL_ and T_CD_. Also a main effect for ‘AHF status’, but no ‘Time’ x ‘AHF status’ interaction related to Intensity-of-arm-hand-use between the affected and non-affected side during uptime was found throughout the rehabilitation phase and post-clinical phase.

#### Mean ratios regarding Intensity of arm-hand use between the affected and the non-affected arm-hand during bimanual activities

Mean ratios regarding Intensity of arm-hand use between the affected and the non-affected arm-hand during bimanual activities for subgroups 1, 2 and 3 are displayed in Fig 6B. The corresponding statistics are presented in Table 4.

As to the ratio of Intensity-of-arm-hand-use between the affected and non-affected side during bimanual activities, similar results were observed as described by the uptime-related ratios above.

## Discussion

The aim of the present study was to assess a) possible improvement or deterioration in actual arm-hand use of sub-acute stroke patients during and after clinical rehabilitation involving a well-defined therapy approach, and b) to what extent actual arm-hand use differs between three subgroups of stroke patients, i.e. patients with either a severely, moderately or mildly affected arm-hand, within this time period. The majority of the study population significantly improved as to Intensity-of-arm-and-hand-use during waking hours (or so-called ‘uptime’). However, when differentiating between unimanual and bimanual task conditions, during the rehabilitation period, i.e. between baseline and clinical discharge, Intensity-of-arm-and-hand-use on the affected side during unimanual activities sharply dropped. In contrast, in the exact same period of time bimanual activities the Intensity-of-arm-and-hand-use on the affected side increased. This indicates that the patients learn to co-use their affected arm-hand more during bimanual skill performance. Duration-of-arm-hand-use on the affected side during bilateral activities improved, especially during the rehabilitation phase, and remained at this higher level during the post-clinical phase. In contrast, Duration-of-arm-hand-use on the affected side during unilateral activities remained at nearly identical levels throughout the rehabilitation and post-rehabilitation phase. These results on actual arm-hand use are in line with improvements observed on both function level and capacity level in the same group as presented by Franck et al. [15].

### Use of the affected hand unimanually

#### Intensity-of-arm-hand-use

In moderately or severely affected patients, the intensity of unimanual arm-hand use on the affected side sharply dropped during the rehabilitation phase, as was also reported by Rand et al., [18]. This phenomenon was less in the mildly affected group. This may have been due to the fact that, during arm-hand treatment, especially the moderately impaired patients are vigorously encouraged to use their affected arm-hand in bimanual activities during their training period in program 2. The mildly impaired patients, are, due to their initial state of impairment, already inclined to work bimanually, using their affected hand less unimanually. A small scale study by Urbin et al.(2015) reported similar results in a group of patients in sub-acute phase comparable with the moderately and mildly affected group as presented in this study. During the post-clinical phase, the intensity of unilateral arm-hand use on the affected side remains low. One year after discharge, patients with a moderately affected arm-hand function achieved and maintained a certain level of intensity of use of the affected hand in unimanual conditions. In contrast, De Niet et al., and Michielsen et al., [54, 55], found that chronic stroke patients hardly use their affected arm-hand unimanually.

Despite a difference in absolute values regarding the Intensity-of-arm-hand-use, relative progressions gained in the moderately affected group in the post-rehabilitation phase did not differ significantly from those in mildly impaired patients. This is interesting, because, in contrast to persons with a mildly impaired arm-hand, patients with a moderately impaired arm-hand first had to regain a substantial level of dexterity as a prerequisite before being able to actually use their affected arm-hand in daily task performance.

#### Duration-of-arm-hand-use

Whereas Intensity-of-arm-hand-use during unimanual activities showed a significant decline during the rehabilitation phase, especially in persons with a mildly and moderately affected hand, Duration-of-arm-hand-use of the affected hand improved in both groups during the exact same period of time. Results achieved during the rehabilitation phase were maintained across the post-rehabilitation phase, which is interesting in particular regarding the moderately impaired group, in which a substantial part of the patients started with no dexterity at all [15]. These results may probably be a consequence of the patient’s learning process to reintegrate the affected hand during skill performance tasks [56]. From the perspective of a patient with a moderately affected hand, gaining confidence in task performance is a powerful issue that may lead to a more positive belief in his/her own capabilities to achieve levels of arm-hand performance previously set out for during goal setting, and may even lead to inter-task transfer of learning towards other, untrained functional tasks [57].

### Use of the affected arm-hand bimanually

#### Intensity-of-arm-hand-use

Regarding bimanual activities, patients with a moderately or mildly impaired hand learned to use their affected hand more frequently and more intensively during the rehabilitation phase and maintained that level till one year after discharge. Similar results were found by Michielsen et al. [55].

Despite an absolute difference in the values regarding Intensity-of-arm-hand-use as measured between the mildly and moderately affected group, the latter group showed a similar pattern of progression in bimanual arm-hand use, both during and after the rehabilitation phase.

To improve and to maintain a certain level of Intensity-of-arm-hand-use in the moderately affected hand used during bimanual task performance is challenging for these patients. They suffer from no or almost no dexterity at the start of the arm-hand rehabilitation process [15]. For these patients it takes courage to use their affected hand in bimanual activities and become *satisfied* about how activities are performed and accomplished [58].

The majority of patients with a severely impaired hand remained unable to use their hand during bimanual performance of tasks in this same period. Progressions observed regarding Intensity-of-arm-hand-use may be explained by: 1) an increase of voluntary movements in flexion synergy in the proximal and/or distal part of the arm; 2) associated movements while moving or performing activities with the non-affected hand or during walking; and 3) performing bimanual exercises as learned to maintain the severely affected arm-hand supple and pain free. However, in contrast to the majority of patients mentioned above who followed program 1, six patients in the severely affected group did show considerable (early) arm-hand improvement. This may be associated with spontaneous recovery. This improvement made them eligible for training in CARAS program 2, whereas all data from the severely impaired patient subgroup were analyzed according to the intention-to-treat principle. A per-protocol analysis of the data (not reported here) leaving out these six patients, showed that in the severely affected group neither Intensity-of-arm-hand-use nor Duration-of-arm-hand-use on the affected side improved significantly across time.

The differences in Intensity-of-arm-hand-use, as observed between the three groups, may be interpreted as follows: 1) In contrast to severely and moderately affected patients, mildly affected patients display voluntary wrist and finger movements at the initial phase of the arm-hand rehabilitation program, associated with a certain degree of cortico-spinal tract integrity [59, 60]. Due to this substantial spontaneous recovery episode, mildly impaired patients become enabled to integrate their affected hand relative more quickly in (bimanual) tasks compared to moderately/severely affected patients. 2) The course of arm-hand rehabilitation of the mildly affected group is less interrupted by problems more commonly seen in the other two groups, like, for instance, presence of cognitive deficits, which could influence motor (re)learning negatively [61]; the appearance of weakness i.e. loss of strength and change in muscle condition [62]; the change in the paretic shoulder’s physical and kinematic properties which results in a less than optimal scapular joint alignment [63]; or swelling or edema of the post-stroke hand[64]. Patients with a moderately affected hand, admitted to CARAS, program 2, went through a considerable development process from not being able to use the affected hand during daily activities at the start of the arm-hand training [15], towards displaying dexterity and a concomitant higher level of Intensity-of-arm-hand-use during rehabilitation and in daily life performance after rehabilitation.

Regarding the moderately and mildly affected group, Uswatte et al., Taub et al., and Liao et al., reported similar findings i.e. a significant increase of Intensity-of-arm-hand-use in sub-acute and chronic stroke patients with a moderately or mildly affected arm-hand who participated in a constrained-induced therapy program [44, 65]. Liao et al., combined functional training with robot practice in mildly impaired chronic stroke patients [66]. However, in contrast to the present study, these studies included relative small study populations with strict inclusion criteria, thus reducing generalizability.

No significant difference were reported by Doman et al., and Rand et al., regarding Intensity-of arm-hand-use in sub-acute stroke patients with a moderately to mildly affected hand, who received arm-hand training [18, 43]. Waddel et al., reported no significant differences in Intensity-of-arm-hand-use in moderately and mildly impaired chronic (≥ 6 months) stroke patients after the rehabilitation phase who participated in a high repetitive, task-specific arm-hand regime [67].

#### Duration-of-arm-hand-use

In moderately and mildly impaired patients improvement in the Duration-of-arm-hand-use of the affected hand during bimanual task performance was observed. However, relative to the non-affected arm-hand, the affected arm-hand continued to play a limited role, which is in line with the studies of Bailey and Michielsen [29, 55].

In mildly impaired patients in the post-rehabilitation period, the non-affected hand is used about one and a half times more than the affected hand. In this same period, patients with a moderately affected hand used their non-affected hand about two and a half times more than their affected hand. Thrane et al., (2011) found a difference in this ratio of up to two and a half times in a group of sub-acute stroke patients with a mildly impaired arm-hand who are comparable with the mildly impaired group as presented in the present study [68]. In contrast, healthy older adults of approximately the same age as the study participants display a more equal Intensity-of-use between both hands [55, 69–71]. Regarding the mildly impaired group the reduction in bilateral arm-hand activity might be due to (a combination of) a lack of motor capability, patient motivation, a shift in routine performance tasks, or a direct result of so-called learned non-use [20, 72].

The moderately affected group as presented in the present study demonstrated far less motor capabilities at the initial start of the rehabilitation phase compared to the mildly affected group [15], which may explain the lower level of bimanual performance in the moderately affected group. Despite this low level, data obtained from the present study suggests that these patients *re-learn* to use their the affected arm-hand during (bi)manual task performance during their rehabilitation period. Within this early, sub-acute phase these patients become enabled to perform a vast number of bimanual tasks in their daily life situation, as learned within program 2 of CARAS. These findings differ from the study of Waddel et al. [67], who reported no improvements in arm hand use in daily performance, after completing an eight-week intensive task-specific arm-hand program. However, their study population consisted of chronic (≥ 6 months post-injury) stroke patients with, on average, a level of arm-hand capacity similar to our moderately affected group measured at clinical discharge. In the post-stroke phase, the moderately affected hand is used in bimanual activities, but still proportionally less than the non-affected hand. The ratio between the affected and non-affected arm-hand remained more or less unchanged during the post-clinical phase. This suggests that patients may have become accustomed to use their affected hand regularly in skill performance in daily situations, a phenomenon also described by other authors [18, 55, 67].

Clinically speaking, sub-acute stroke patients who followed an arm-hand training featuring CARAS, program 2 and program 3, improved on Intensity-of-use and Duration-of-use of their moderately or mildly affected arm-hand.

### Considerations

This study is not without limitations. First, the inability to differentiate between signals stemming from task-specific (e.g. reaching or grasping) or non-task-specific arm-hand movement may limit the interpretation of results regarding any qualitative aspect of arm-hand motor behavior [30]. Wrist accelerometry is a valid and reliable measurement method [20, 31]. However, it measures ‘use’ and not ‘purposeful use’[73], and does not provide information about movement quality or specific activities that were performed during the wearing period. Therefore, data on actual arm-hand use provided in this study have to be interpreted with some care when translating these results towards real arm-hand skill performance. Techniques to identify (the quality of) specific among multiple activities using body worn sensors are upcoming and are promising tools to further improve actual arm-hand skill performance measurement [74].

Secondly, data on actual arm-hand use, based on accelerometry, depend on the way how arm-hand use has been defined, and on how data are collected and processed [20]. The term ‘counts’, as a measure of ‘amount of use’ [23, 75], is often used. However, the calculation of ‘counts’ is not always the same across studies. Therefore, in order to be able to compare our results with other studies, in the present study it is explicitly formulated how the ‘amount of use’ metric was calculated, based on the raw accelerometer data of the Actiwatches used. This makes careful comparisons with other studies regarding the use and interpretation of accelerometry data possible.

Thirdly, in the present study an intention-to-treat analysis has been performed, which may have resulted in substantial larger within-group variances in the three subgroups.

In the severely affected group some patients showed substantial large progressions regarding Intensity-of-arm-hand-use during the clinical rehabilitation phase, and were transferred from CARAS program 1 to CARAS program 2 during the rehabilitation phase. These improvements in a subpopulation in the severely affected subgroup could explain the statistically significant differences being found. A per-protocol analysis of the data (data not reported in this paper) revealed that in the remainder of participants in subgroup 1 neither a statistical nor a clinically relevant improvement in actual arm-hand use was found.

Fourthly, contrary to participants from subgroup 1 and 3, patients with a moderately affected arm-hand received a second six weeks period of training in order to experience functionally meaningful progressions in the use of their affected hand and the possibility to use their affected arm-hand more purposefully in bimanual skill performance.

Fifthly, in the present study a distinctly low proportion of female participants participated (see Table 1), especially in subgroup 2 and 3. This may have influenced our results. In general, most instrumental activity of daily living (IADL) tasks which require arm-hand usage are traditionally done by women. Though physically capable to perform IADL tasks, men often rely on their spouses performing IADL tasks like cooking and laundry [76]. However, despite this unequal representation of gender across both groups, group results indicate progression in both ‘intensity-of-arm-hand-use’ and ‘duration-of-arm-hand-use’. Subgroup 1 contained proportionally more female participants compared to subgroup 2 and subgroup 3. Therefore, gender differences most likely had even less influence on study results regarding the latter group.

Sixthly, the average post-stroke time at point of admission to the study of patients of subgroup 1 differed from the average post-stroke time in patients admitted to subgroup 2 and 3 (see Table 1). This is a phenomenon typically seen in clinical situations in the sub-acute phase after stroke. In contrast to moderately and mildly impaired patients, who were trained to increase intensity and duration of use of their affected hand, patients of subgroup 1 were encouraged to keep their severely affected arm in good condition instead of being stimulated, to no avail, to regain arm-hand use [14]. This has led to patients from different groups to improve at a different rate, leading to between-group differences. Another source of between-group differences is, of course, the aforementioned difference in stroke severity at entry into the study.

## Supporting information

S1 Table (SAV)
